# Reflection on modern methods: when is a stepped-wedge cluster randomized trial a good study design choice?

**DOI:** 10.1093/ije/dyaa077

**Published:** 2020-05-09

**Authors:** Karla Hemming, Monica Taljaard

**Affiliations:** d1 Institute of Applied Health Research, University of Birmingham, Birmingham, UK; d2 Clinical Epidemiology Program, Ottawa Hospital Research Institute, Ottawa, ON, Canada; d3 School of Epidemiology and Public Health, University of Ottawa, Ottawa, ON, Canada

**Keywords:** Cluster randomized trial, stepped-wedge, design justification

## Abstract

The stepped-wedge cluster randomized trial (SW-CRT) involves the sequential transition of clusters (such as hospitals, public health units or communities) from control to intervention conditions in a randomized order. The use of the SW-CRT is growing rapidly. Yet the SW-CRT is at greater risks of bias compared with the conventional parallel cluster randomized trial (parallel-CRT). For this reason, the CONSORT extension for SW-CRTs requires that investigators provide a clear justification for the choice of study design. In this paper, we argue that all other things being equal, the SW-CRT is at greater risk of bias due to misspecification of the secular trends at the analysis stage. This is particularly problematic for studies randomizing a small number of heterogeneous clusters. We outline the potential conditions under which an SW-CRT might be an appropriate choice. Potentially appropriate and often overlapping justifications for conducting an SW-CRT include: (i) the SW-CRT provides a means to conduct a randomized evaluation which otherwise would not be possible; (ii) the SW-CRT facilitates cluster recruitment as it enhances the acceptability of a randomized evaluation either to cluster gatekeepers or other stakeholders; (iii) the SW-CRT is the only feasible design due to pragmatic and logistical constraints (for example the roll-out of a scare resource); and (iv) the SW-CRT has increased statistical power over other study designs (which will include situations with a limited number of clusters). As the number of arguments in favour of an SW-CRT increases, the likelihood that the benefits of using the SW-CRT, as opposed to a parallel-CRT, outweigh its risks also increases. We argue that the mere popularity and novelty of the SW-CRT should not be a factor in its adoption. In situations when a conventional parallel-CRT is feasible, it is likely to be the preferred design.

## Background

**Figure 1:**
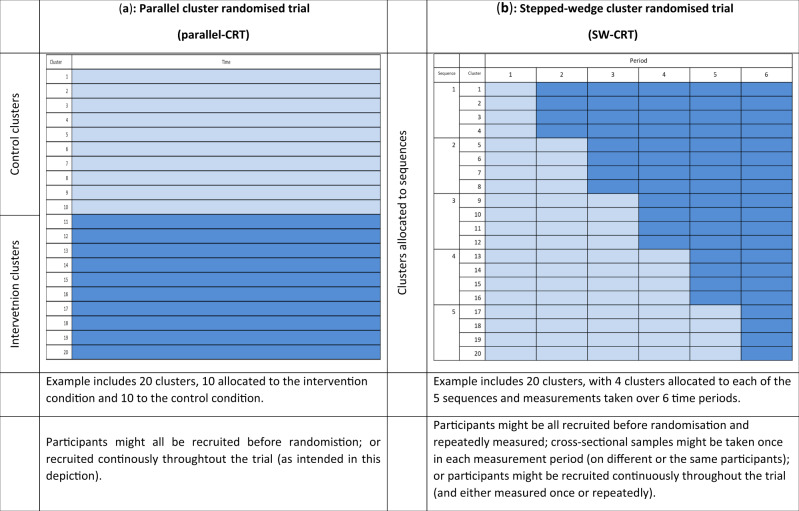
Schematic illustration of the parallel cluster randomized trial and the stepped-wedge cluster randomsed trial. (a) Parallel cluster randomized trial (parallel-CRT); (b) stepped-wedge cluster randomized trial (SW-CRT).

The cluster randomized trial is a firmly established study design particularly useful for pragmatic evaluations of health policy interventions, such as changes to the way services are delivered, educational interventions or public health type interventions, to name but a few.^1–3^ In a parallel cluster randomized trial (parallel-CRT) half the clusters (such as hospitals, wards or communities) are randomly assigned to the intervention condition and half to the control condition (Figure 1a). The stepped-wedge cluster randomized trial (SW-CRT) involves the sequential transition of clusters from control to intervention conditions in randomized order, until all clusters are exposed (Figure 1b).^4^ The use of the SW-CRT is growing rapidly, from just a handful of published reports at the beginning of the century to 30 to 40 protocols per year today.^5–7^ Yet, it is under-appreciated that the SW-CRT is at greater risks of bias compared with the conventional parallel-CRT. This is because in an SW-CRT, observations under the control condition are collected at a systematically earlier calendar time compared with those under the intervention condition. Unlike any other randomized design, which seeks to minimize confounders, the SW-CRT therefore induces a confounder by design. Furthermore, the SW-CRT may be subject to greater risks of other biases compared with a conventional parallel-CRT.^8^ For this reason, the CONSORT extension for SW-CRTs requires that investigators provide a clear justification for using this design.

Unfortunately, despite these increased risks of bias, the use of the SW-CRT is increasing rapidly and the mere popularity and novelty of the SW-CRT seems to be a factor in its adoption. This has potential ramifications for evidence-based medicine and policy decisions, because the robustness of the evidence base on which these decisions are made will become questionable should researchers start to use the SW-CRT when a parallel-CRT would have been a more robust alternative. This situation has been exacerbated by the confusion in the literature as to what are the appropriate justifications for using this design. Some authors have attempted to dispel the myths around the apparent benefits of the design, especially those pertaining to ethics and logistical constraints.^9–11^ Nonetheless, the most commonly cited reasons for choosing an SW-CRT are its perceived logistical, social and ethical benefits.^7^ In this paper we therefore not only outline the potential conditions under which an SW-CRT might be an appropriate choice, but provide a clear narrative on why and how the design is at risk of bias, as a way of underscoring the importance of a careful justification for use of the design. Our objectives are to provide researchers with recommendations for when the SW-CRT is a good study design. We limit our consideration to the evaluation of interventions which cannot easily be withdrawn from practice once implemented: if withdrawals were possible, many other alternative designs would have to be considered.

## Risks of bias in an SW-CRT

Risks of bias in randomized trials have been carefully described in the Cochrane Systematic Review Risk of Bias tool (RoB2),^12^ and an adaptation of the main guidance has been made for parallel cluster trials.^13^ We have mapped these risks across both parallel-CRT and SW-CRT study designs, to highlight differences in risks of bias, and we discuss each of these risks in detail below. Some of these risks appear likely to be greater in SW-CRTs, and these are summarized in Table 1. Other risks of bias documented in RoB2, but not considered as important in the differential choice between the designs, are summarized in Supplementary Table 1, available as Supplementary data at *IJE* online. We have used the terms SW-CRT and parallel-CRT in a very broad sense, but acknowledge that these studies can be designed and implemented in very different ways. For example, parallel-CRTs can be conducted so that individuals are recruited and clusters then randomized; or clusters might be randomized and individuals subsequently recruited, possibly extended over long periods^3^ (Figure 1). Not all parallel-CRTs and SW-CRTs will thus be at equal risk of bias, and we have highlighted where necessary when different forms of designs influence this risk.

**Table 1.** Risks of bias in the stepped-wedge cluster randomized trial (SW-CRT) with emphasis on the comparative risks compared with a parallel cluster randomized trial (parallel-CRT)

Risk of bias	Description	Parallel-CRTs	SW-CRTs	Mitigation methods
Bias arising from the staggered nature of the roll-out (analytical biases)	In an SW-CRT, the observations taken under the control condition are from a systematically earlier calendar time compared with the intervention condition. This means that calendar time is a potential confounder. Therefore a model based analysis must be used to differentiate changes in the outcome attributable to the intervention from those attributed to secular changes. Analytical biases refer to bias in the estimation of treatment effects and confidence intervals because of misspecification of this statistical model. In an SW-CRT, if the model for the secular change is misspecified, the study will be at risk of analytical bias.	CRTs run at a single cross-section, are not at risk of time imbalances and time does not need to be allowed for in any model-based analysis. CRTs are however at risk of other biases due to model misspecification, and this is particularly the case when there are a small number of clusters, or when the underlying correlation structure is misspecified	SW-CRTs are at risk of biases due to model misspecification when the underlying secular trend is misspecified; when there are a small number of clusters; or when the underlying correlation structure is misspecified	SW-CRTs with a large number of clusters and adequate randomization methods may be robust to assumptions of common secular trends. Avoid assuming strong parametric assumptions for secular trends (allowing for non-linear effects). Small sample corrections and time-dependent correlation structures are also important. Sensitivity analysis for any model-based assumptions, such as the assumption that all clusters follow the same secular trend
Bias arising from identification or recruitment of individual participants within clusters	When identification and recruitment of participants occurs with knowledge of the trial arm or sequence, this can lead to differential recruitment and identification between arms or sequences. This bias is described in RoB2 for cluster trials under Domain 1b	In parallel-CRTs, it is sometimes possible to recruit participants before randomization. CRTs recruiting after randomization, without broad eligibility criteria and without taking steps to conceal the allocation from the person responsible for recruiting into the study, will be at risk of identification and recruitment biases	SW-CRTs conducted using cross-sectional designs (in which different participants are measured on each measurement occasion), without broad eligibility criteria and without taking steps to conceal the allocation from the person responsible for recruiting into the study, will be at risk of identification and recruitment biases. In SW-CRTs, using cohort designs (in which each participant is repeatedly measured throughout the trial), recruiting after randomization without broad eligibility criteria and without taking steps to conceal the allocation from the person responsible for recruiting into the study, will be at risk of identification and recruitment biases	Recruit participants before randomization; have broad eligibility criteria; take steps to conceal the allocation from the person responsible for recruiting. Where ethically appropriate, take a complete enumeration of outcomes from the entire cluster
Bias due to within cluster contamination	Trials which intend to measure the effect of offering treatment in everyday practice are unlikely to be conducted with blinding of the participant to their allocation status. Deviations from the intended intervention can occur if those in the control condition receive the intervention condition (or vice versa)—referred to here as contamination. This bias is described in RoB2 under Domain 2	Contamination across arms in a parallel-CRT is unlikely assuming the cluster unit has been chosen appropriately to avoid contamination	SW-CRTs are at additional risk of contamination across treatment conditions when: either the intervention condition takes longer to embed in practice than planned; or when there is a delayed assessment of outcome in a sample with long exposure to the intervention condition	Inclusion of a transition period to allow an intervention to be fully embedded before data are collected. When participants have a long exposure to the intervention, delayed assessments of outcomes should be avoided
Additional concerns				
Chance imbalance	Although not a bias, randomization of a small number of units can create an imbalance in prognostic characteristics across treatment groups. These chance imbalances might mean that it can become difficult to attribute any differences in the outcome to the treatment and the study might lack face validity. Restricted randomization procedures can help mitigate this risk	In parallel-CRTs with a small number of clusters, the randomization might not lead to a similar distribution of characteristics across the two arms	In SW-CRTs with a small number of clusters, the randomization might not lead to a similar distribution of characteristics across the sequences, but time-invariant characteristics are likely to be well balanced across the treatment conditions	Include a large number of clusters. Use restricted randomization procedures on well-measured prognostic variables

### Bias arising from the staggered nature of the roll-out

An important consideration in an SW-CRT is the choice of analytical approach to ensure unbiased estimates of the intervention effect. Accurate estimates are needed of both the treatment effect and its standard error. The analysis of SW-CRTs is complicated by the fact that underlying changes over time—called secular trends—may be partially confounded with the intervention effect: thus, an apparent effect due to the intervention may in fact be due to natural changes over time. This means that mathematical modelling is needed to disentangle changes in outcomes due to secular trends from changes in outcomes due to the intervention.^14^ These models often make the assumption that any underlying secular trends are the same across all clusters.^15^ Trials conducted across several different countries, with a few clusters in each country, for example, are unlikely to meet these assumptions. A parallel-CRT (conducted with a one-off randomization or randomization in batches) is not at risk of time-varying confounding because the design is balanced on time. SW-CRTs randomizing a large number of clusters are unlikely to be at risk of this bias.

The SW-CRT faces other challenges due to its longitudinal nature. Observations collected under the intervention condition consist of a mixture of observations collected shortly after the roll-out of the intervention and observations collected some time after the roll-out of the intervention. In any analysis, these observations are usually pooled and so the estimated treatment effect becomes a time-averaged effect. Furthermore, observations in an SW-CRT are taken repeatedly through time, so that within-cluster correlations might take a more complicated form than a simple exchangeable structure.^16–18^

There are therefore several assumptions typically made at the analysis stage: functional form of the time trend, homogeneity of this time trend across clusters, and estimation of a time-average treatment effect. Analysis methods that do not appropriately adjust for secular trends or do not account for complex within-cluster correlations provide biased estimates of treatment effects and their standard errors.^19^^,^^20^ Parallel-CRTs, conducted at a single cross-section, do not have these additional complexities in the analysis.

### Identification and recruitment biases

Identification and recruitment bias refers to bias arising from recruiting or obtaining data on selectively different samples in the intervention and control periods of the study. Identification and recruitment bias is a particular concern in those cluster-randomized trials where participants are identified and recruited after randomization.^12^^,^^21^ This opens up the possibility that identification and recruitment take place with knowledge of the treatment condition to which the cluster has been randomized. In parallel-CRTs, empirical evidence shows that this can lead to differential recruitment between study arms.^22^^,^^23^ To our knowledge, no studies have empirically examined risks of identification and recruitment bias in SW-CRTs. However, the implications of these biases in SW-CRTs are more difficult to assess than in parallel-CRTs, because of the potential influence of not only the knowledge of the intervention at the time of recruitment, but the influence of the knowledge of when the transition to the intervention will occur.

There are recommended strategies to mitigate identification and recruitment biases, and these include minimizing the number of eligibility criteria and recruitment by someone independent of the trial, who is blind to cluster status.^24^^,^^25^ Recruitment biases may be avoided entirely in cases where it is ethically appropriate to include data from all cluster members without their prospective recruitment and consent, i.e. when a research ethics committee grants a waiver of informed consent.^26^

### Within-cluster contamination

Within-cluster contamination refers to biases due to collected data under the control condition becoming contaminated by the intervention condition (or vice versa). The unit of clustering in a parallel-CRT is chosen, in part, so that observations under the control condition will not inadvertently be exposed to the intervention condition. Yet, within-cluster contamination may be more likely to occur in an SW-CRT since every cluster is exposed to both control and intervention conditions. For example, a provider in a site that is still in the control condition may deliberately or inadvertently implement the intervention before the allocated time (control condition is contaminated by the intervention condition). Conversely, a provider in a site that has already crossed to the intervention condition may deliberately or inadvertently persist in applying the control intervention (intervention condition is contaminated by the control condition).^27^^,^^28^

Within-cluster contamination might also arise at the level of the individual. In a setting where participants have a long exposure to the trial (e.g. patients in intensive care units where some patients have a prolonged stay and may still be in the intensive care unit at the time of crossing over), it is possible that observations from individuals included in the control condition become contaminated with the intervention condition. In trials where participants have a short exposure to the trial (e.g. in the so-called continuous recruitment short exposure design), this type of contamination is unlikely to arise.

There are again strategies to minimize the impact of within-cluster contamination by design.^28^ These include the inclusion of a transition period to allow an intervention to be fully embedded before data are collected under the intervention. In other situations, for example where participants have a long exposure to the intervention, delayed assessments of outcomes should be avoided.

### Chance imbalances

Although not a bias, chance imbalance in any randomized trial is important. In a parallel-CRT, randomization of an adequate number of clusters should create a balance in known and unknown prognostic characteristics across treatment groups. However in practice, particularly in studies with a small number of clusters, chance imbalances can occur. These chance imbalances might mean that it can become difficult to attribute any differences in the outcome to the treatment and the study might lack face validity. In designs such as the SW-CRT where every cluster is observed under both control and intervention conditions, these chance imbalances are likely to be less important. Yet, SW-CRTs are not immune from these imbalances: the imbalance might exist across sequences (Figure 1)—so that for example, those clusters randomized to transition early in the study are different from those which transition late in the study. Constrained randomization using key cluster-level characteristics can prevent imbalance on cluster-level characteristics;^29–31^ yet, these methods require knowledge and availability of important prognostic factors before study commencement.

## Broad justifications for the use of an SW-CRT design

SW-CRTs are subject to several risks of bias that might challenge the strength of the evidence generated from this design. Some of these risks of bias may affect other types of cluster randomized designs too, but many appear to be greater under the SW-CRT. We therefore contend that the use of the SW-CRT must be justified. We outline several situations that might provide support for undertaking an SW-CRT. It is not our intention to suggest that these are hard and fast justifications, but simply that these are considerations which, especially when taken together, may support adopting an SW-CRT. In Supplementary Boxes 1 and 2, available as Supplementary data at *IJE* online, we consider whether and how these justifications apply for a recently funded SW-CRT evaluation of a new health policy intervention in kidney transplantation in the UK, and consider which risks of bias may apply given that an SW-CRT design was ultimately used.^32^

### Justification 1: the SW-CRT provides a means to conduct a randomized evaluation

Interventions are frequently rolled out without any robust randomized evaluation. Sometimes the roll-out might be sequential because of a limited resource or capacity to roll out to the entire health system simultaneously, or because a gradual implementation allows the possibility to learn from earlier implementation in such a way that the intervention is adapted as more is learned. Here, if stakeholders can be convinced to randomize the order of the roll-out, using the SW-CRT might provide a means to both obtain a robust evaluation and allow staggering of the roll-out. These might provide sufficient justifications for using an SW-CRT design when any alternative evaluation is a non-randomized evaluation (i.e. before and after study) or no evaluation.^9^ However, if stakeholders can be convinced of the benefits of randomizing the order of the roll-out to align with an SW-CRT, then it might also be possible to convince the stakeholders of the benefits of a parallel-CRT conducted in such a way so that all of the clusters receive the intervention at the end of the evaluation (i.e. a waitlist design), if the parallel-CRT provides evidence of effectiveness.

### Justification 2: the SW-CRT facilitates cluster recruitment

Cluster randomized trials often obtain permission from individuals called ‘gatekeepers’ who can allow cluster participation in the trial.^26^^,^^33^ Examples of gatekeepers are general practice managers, ward matrons and lead consultants. These gatekeepers likely have to obtain nominal support from other cluster stakeholders (such as other nurses, GPs or consultants or members of society). Sometimes, gatekeepers or stakeholders might be reluctant to participate in a trial unless they are guaranteed to have the opportunity of receiving the intervention (which might be a novel programme or system intervention expected to offer some benefits). This can arise because of a general awareness of the need for improvement and the expectation that the intervention is better than no intervention. Even if the intervention is offered at the end of the trial (i.e. waitlist design), this is often not considered as satisfactory as receiving it during the trial, perhaps because of the effort involved in data collection which can be offset by the perceived benefit of participating in a novel intervention. These desires and a priori beliefs might mean that stakeholders are more likely to participate in the trial when designed as an SW-CRT. Enhanced cluster recruitment in the SW-CRT is therefore sometimes put forward as a justification for using the SW-CRT over the parallel-CRT.^9^^,^^34^ To serve as a legitimate justification for adopting an SW-CRT, researchers could attempt to demonstrate that clusters are indeed more likely to participate in an SW-CRT trial after being fully informed about alternatives such as a parallel-CRT and waitlist designs.

### Justification 3: the SW-CRT creates a logistically feasible design

Sometimes the roll-out of an intervention might necessarily be sequential because of a limited resource or capacity to roll out to the entire health system simultaneously. In these situations, the SW-CRT might be feasible and can sometimes be justified based on needing to stagger the roll-out for logistical reasons.^9^^,^^34^ However, a parallel-CRT can also be conducted in a staggered way and so is not necessarily infeasible under these logistical constraints.^35^ In a staggered parallel-CRT design, allocations take place in ‘batches’ or blocks of time. A parallel-CRT only becomes infeasible if the roll-out of the intervention is constrained to only a couple of clusters simultaneously. It should also be noted that the sequential roll-out in an SW-CRT can bring about its own logistical issues,^9^^,^^34^ for example organizing research ethics approvals for all centres in advance of the trial, and ensuring that all centres are ready to implement the intervention according to the allocated schedule can be challenging.^36^

### Justification 4: the SW-CRT has increased statistical power

The number of available clusters may be restricted based either on availability, willingness to participate or limited trial budgets. In these circumstances, an SW-CRT may achieve the desired power with fewer clusters than a parallel-CRT.^37^ In fact, with a small number of clusters, 80% or 90% power might not even be achievable in a parallel-CRT, whereas an SW-CRT can achieve 80% or 90% power with the same number of clusters (see Supplementary Material 1, available as Supplementary data at *IJE* online). This is particularly the case when the cluster size and/or the intra-cluster correlation is high.^38^ Whether an SW-CRT is more powerful than a parallel-CRT needs to be determined on a case-by-case basis considering the competing requirements of how many clusters are available, the sizes of the clusters and outcome type. However, general rules of thumb are that the SW-CRT will likely require fewer clusters than a parallel-CRT when any of the following are true: the outcome is such that it has a high intra-cluster correlation (e.g. the clusters are all very dissimilar or the outcome is a binary variable with high prevalence^39–41^) and the cluster sizes are large. These increases in power achievable under the SW-CRT (when the intra-cluster correlations are high) are due to the within-cluster comparisons inherent in the SW-CRT, and are related to the benefits that the SW-CRT can provide in terms of reducing the imbalance on cluster-level characteristics across treatment conditions. This justification is strongly related to economic trial cost efficiency and total study duration (below), and might thus be justified from the perspective of trial costs rather than power.

### Other considerations

#### Study duration

An additional consideration in choosing between a parallel-CRT and an SW-CRT is the overall study duration and whether there is an imperative to provide an evaluation of the intervention’s effectiveness in a shorter amount of time. Whether the SW-CRT or parallel-CRT will take a shorter-duration depends on the specific circumstances of the trial.^42^ For example, if there is an adequate number of clusters available and the randomization is once-off rather than in batches, it is possible that the parallel-CRT can be completed in a shorter duration, although it might require a larger number of clusters (see Supplementary Box 2 for an example, and Supplementary Material 1, available as Supplementary data at *IJE* online). Inherently, the SW-CRT is a repeated measures design and the total study duration is a function of both the number of steps and the duration of each step.

#### Time to realize the effect of intervention

Study designs may need to allow time for the intervention to start working and affect outcomes.^4^ In the evaluation of non-complex interventions, this is usually relatively straightforward (e.g. a drug is given to a patient and a patient is thus exposed). However, in the evaluation of complex interventions it might take considerable time for an intervention to become fully embedded in the setting and influence outcomes. Transition periods can be incorporated to allow for this delay. In some settings, transition periods might need to be quite long which can increase the duration of the SW-CRT over a parallel-CRT.

#### Common myths about the design

There have been several common myths about the SW-CRT.^9–11^ Some of these myths suggest positive benefits of the SW-CRT and others suggest negative benefits or contraindications. Those that are often used to suggest that the SW-CRT brings about positive benefits include that the SW-CRT is ethically appropriate when the intervention is expected to do more good than harm. Other myths are related to why the SW-CRT might not be a good choice and these include that the SW-CRT will expose more or fewer participants to an intervention of unknown effectiveness. These myths are expanded and expelled in Table 2.

**Table 2.** Common misconceptions about the pros and cons of a stepped-wedge cluster randomized trial (SW-CRT)

Misconception	Description	Rebuttal
Pros		
The SW-CRT is ethically appropriate when the intervention is expected to do more good than harm	One justification of the SW-CRT design, that is often put forward, contends that the SW-CRT is appropriate when there is an ethical imperative for all clusters to receive the intervention, or perhaps where it is expected that the intervention will likely do more good than harm^7^^,^^11^	Justification for a randomized evaluation is clearly necessary when using any form of randomized trial design, but is actually very important in what follows. If a randomized evaluation is justified, i.e. when clinical equipoise holds, then it becomes reasonable to expose some clusters to the intervention condition but not others, so that robust evidence can be generated. Likewise, it is also reasonable to withhold the intervention from some individuals. However, if the intervention is known to be effective, then there is no ethical justification for withholding the intervention from some clusters or some individuals. Consequently, if it is argued that there is an ethical imperative for all clusters to receive the intervention, the same argument should mean that there is an ethical imperative for all individuals to receive the intervention without delay. Yet, in an SW-CRT, only half of participants (in a cross-sectional design) will receive the intervention and for many there will be a delay before receiving it. There is thus no ethical benefit of using the SW-CRT, although its use can be ethical in situations of clinical equipoise^11^^,^^34^
Cons		
The SW-CRT exposes more clusters or more individuals to an intervention of unknown effectiveness or potential harm	The SW-CRT is commonly perceived to increase the number of individuals or clusters exposed to an intervention of unknown effectiveness, or even, potential harm^10^	In an SW-CRT, all clusters are ultimately exposed to the intervention condition. However, this does not necessarily mean that an increased number of clusters will be exposed compared with how many would have been exposed under a parallel CRT. This is because it might be the case that in a parallel-CRT, more than twice the number of clusters are needed to achieve the same power.^37^ In the same way, neither does an SW-CRT necessarily increase the number of individuals exposed to the intervention.^37^ For example, a standard SW-CRT with a cross-sectional design will expose only half of the participants to the intervention (see Figure 1 for an explanation)

## Discussion

The SW-CRT is a novel randomized design which has the potential to facilitate the robust evaluation of health policy and other interventions. However, the SW-CRT is more complicated in its design and analysis.^27^^,^^28^ The means that there is an increased risk that the study might fail to deliver on its objectives and/or produce misleading conclusions. For these reasons, the use of the SW-CRT must be carefully justified and alternative designs considered when they are both feasible and more robust. There are other factors for consideration, which we have not touched upon here. These include the possibility that the SW-CRT can provide data to examine differential treatment effects across clusters^43^^,^^44^ and investigate if treatment effects change over time.^43^

We have considered and commented on various commonly proposed justifications for using the SW-CRT.^9^ These include: (i) the SW-CRT provides a means to conduct a randomized evaluation which otherwise would not be possible; (ii) the SW-CRT facilitates cluster recruitment as it enhances the acceptability of a randomized evaluation either to cluster gatekeepers or to other stakeholders; (iii) the SW-CRT is the only feasible design, due to pragmatic and logistical constraints (for example the roll-out of a scare resource); and (iv) the SW-CRT has increased statistical power over other study designs (which will include situations with a limited number of clusters). As the number of arguments in favour of an SW-CRT increases, the likelihood that the benefits of using an SW-CRT, as opposed to a parallel-CRT, outweigh its risks also increases. We have also argued that the SW-CRT might increase the overall duration of the study, and this might be of particular importance when the effect of the intervention is expected to take some time to materialize.

## Supplementary Data

Supplementary data are available at *IJE* online.

## Funding

This research was partly funded by the UK NIHR Collaborations for Leadership in Applied Health Research and Care West Midlands initiative. KH is funded by an NIHR Senior Research Fellowship SRF-2017-10-002. This research is independent of the funder.

## Conflict of Interest

None declared.

## Supplementary Material

dyaa077_Supplementary_Data
