# Supplementary material for: Reflection on modern methods: when is a stepped-wedge cluster randomized trial a good study design choice?
Source: Int J Epidemiol. 2020 May 9;49(3):1043–52. doi: 10.1093/ije/dyaa077 (PMC7394949; doi:10.1093/ije/dyaa077)
Supplement: dyaa077_Supplementary_Data [file dyaa077_supplementary_data.docx]

**Supplementary Material**

| **Box S1: Illustrative case study – the PITHIA Trial risk of bias under a SW-CRT** |
| --- |
| The UK National Institute for Health Research has recently funded a SW-CRT evaluation of a biopsy service to improve outcomes in kidney transplants [Ayorinde 2019]. This is a randomised evaluation of a service that has been implemented in one transplant centre for ten years. Every four months a randomly-chosen group of transplant centres will be offered access to the biopsy service. By the end of the trial, all 22 transplant centres in the UK will have access. The trial will use routinely collected data. Researchers needed to identify which study design, i.e. a parallel-CRT or SW-CRT, was going to be more robust to potential barriers to success; and provide an appropriate justification for the choice. We consider each of these risks in turn and rate them as low, moderate or high risk under a SW-CRT design. |
| *Analytical biases (moderate to high risk):* The primary risk which a SW-CRT, with a small number of clusters, has to contend with, unlike a parallel-CRT, is the risk of bias due to misspecification of underlying temporal trends. Whilst there is no immediate reason to suspect that clusters in the PITHIA trial will follow different secular trends (an assumption of almost all model based analyses), inferences from the trial will have to rely on mathematical modelling and the modelling of trends will be subject to bias due to misspecification. |
| *Chance imbalance (moderate to high risk):* Whilst randomised, the relatively small number of clusters in the PITHIA trial increases the risk of imbalance to some degree on some characteristics. Under a SW-CRT this might mean for example, all small transplant centres might be randomly allocated to transition to the service early in the design. This would still be a risk under a parallel-CRT, where chance randomisation might mean all small transplant centres were allocated to the intervention condition for example. This risk can be mitigated in part by using a constrained randomisation procedure. |
| *Identification and recruitment biases (low risk):* Given that the data are routinely collected in the PITHIA trial and the intervention was a change to the services provided; a research ethics board approved a design in which participant consent was not obtained. The study (irrespective of SW-CRT or parallel-CRT) is thus protected from recruitment bias; furthermore, the trial has broad and objective eligibility criteria and is therefore likely to be protected from identification biases. |
| *Randomisation bias (low risk):* Under a SW-CRT design there may be risks of bias due to failure to conceal the timing of the cross-over from control to the intervention condition from providers. In PITHIA some attempt was made to mitigate this by revealing the date of the cross-over no earlier than three months before the transition (time needed to allow providers to manage the change to intervention condition). |
| *Within-cluster contamination (low to moderate risk):* Given the complex nature of the intervention: a roll-out of a biopsy service and education to promote its use, the PITHIA trial should ideally should have allowed for a transition period (although one was not included). Observations collected under the intervention condition in the time immediately after roll-out might therefore not be fully exposed to the intervention. On the other hand, given patients are exposed to the study interventions for a very short period and the fact that the intervention is not available outside the trial, there are likely to be minimal concerns due to within-cluster contamination of control with intervention condition. |

| **Box S2: Illustrative case study – justification for choosing a SW-CRT in the PITHIA Trial** |
| --- |
| The PITHIA trial opted to use a stepped-wedge cluster randomised design. We consider if this choice is justified. |
| *Justification 1: The SW-CRT provides a means to conduct a randomised evaluation which otherwise would not be possible;* |
| In the setting of the PITHIA trial, there was no plan to roll-out this intervention to all transplant units in the UK outside of the trial setting and there was thus no need to distribute a scarce resource. In the PITHIA trial, it would therefore not be appropriate to justify the use of the SW-CRT design on the grounds that the intervention was to be rolled-out irrespective of any trial evaluation. |
| *Justification 2: The SW-CRT enhances the acceptability of a randomised evaluation* |
| With reference to the PITHIA trial, in the single centre where the intervention had already been used, a beneficial effect had been observed. Whilst an effect in a single centre is no guarantee of effectiveness, it might lead key consultants (gatekeepers) and any patients who might have had a say in whether the centre participated in the trial (stakeholders) to hold the view that the intervention was desirable. In the PITHIA trial, all clusters agreed to participate. Whether this would have been the case under a parallel design is an unknown counterfactual. |
| *Justification 3: The SW-CRT creates a logistically feasible design* |
| In the PITHIA trial, the delivery of the intervention involved training and the initial introduction of the intervention had resource implications. As the team of experts involved in the delivery of the training and roll-out of the intervention was small, they lacked capacity to roll out the intervention to more than a handful of clusters simultaneously. The SW-CRT therefore creates a logistically feasible design. However, a design that might have been feasible is a parallel-CRT design in which at each of several randomization points, a subset of the clusters are allocated to the intervention or control conditions. |
| *Justification 4: The SW-CRT has increased statistical power* |
| In the PITHIA trial, there are only 22-transplant units (clusters) in the UK. Excluding the one transplant unit which had already rolled-out the intervention, there were therefore a maximum of 21 clusters available for inclusion. The study was also limited to a maximum cluster size constrained by the number of eligible kidneys over a 24-month period. Under realistic sample size assumptions, 90% power would not have been achievable under a parallel-CRT, but was achievable under a SW-CRT [Supplementary material 1 and Ayorinde 2019]. For the PITHIA trial it was therefore the case that the SW-CRT was a more statistically powerful design. |
| *Other considerations: Study duration* |
| In the PITHIA trial, the stepped-wedge time intervals were of 4-month duration and there were 6-intervals (requiring a duration of 24 months). On the other hand, a parallel-CRT design could have been completed in a single 4-month interval, but would have required 64 clusters, which were not available in this situation (Supplementary material 1). The SW-CRT therefore does not make the study of longer duration compared to the parallel design. |
| *Other considerations: Time to realise the effect of the intervention* |
| In the PITHIA trial the implementation of the new biopsy service was expected to be almost instantaneous, although it might be expected to take some time for service users (doctors) to fully appreciate its availability. This time needed to embed the intervention could have been estimated and transition period included to ensure that observations collected under the intervention condition have been properly exposed to the new service. |
| *Other considerations: Myths* |
| In the setting of the PITHIA trial, there was no plan to roll out this intervention to all transplant units in the UK outside of the trial setting and there was no strong evidence of effectiveness. There was thus no need to ensure all transplant units received the intervention. Furthermore, it was also not the case that a reason for not adopting the SW-CRT was that it would exposure more people to an intervention of unknown effectiveness: it did require all 22 transplant units to become exposed but under a parallel design with only 22 transplant centres included would not have achieved 90% power. |

| **Table S1: Risks of bias in parallel-CRT and SW-CRT study designs for less relevant domains in RoB2** | | | |
| --- | --- | --- | --- |
| **Domain** | **Description** | **Parallel-CRTs** | **SW-CRTs** |
| Bias arising from the randomisation process | Randomisation refers to the process of allocating clusters to arms (in a parallel-CRT) or to sequences (in a SW-CRT). Biases can arise if this allocation is not random or is not adhered to (at the level of the cluster). This bias is described in RoB2 under Domain 1a. | Provided the randomisation is conducted by an independent person and it takes place after all clusters have agreed to participate, the randomisation of clusters to arms is unlikely to be subverted in a parallel-CRT. In parallel-CRTs randomisation can sometimes be performed in batches of clusters ready to start the trial and this does not usually induce any bias. | In SW-CRTs, so that all clusters have an equal chance of being allocated to all sequences, randomisation should be performed after ALL clusters have agreed to participate, otherwise biases might arise. The timings of transitions might be concealed until just before transition or it might be revealed at start of study. Any risks of bias due to early or late revealing of transition dates is uncertain and could vary in different trials. |
| Bias due to missing outcome data | Missing outcome data often occurs in randomised trials. Where the missingness is differential across arms or sequences, this can cause bias in estimation of treatment effects. Missingness can be differential across arms or sequences even when the proportion missing is similar across arms (for example when missingness is dependent on prognostic factors). This bias is described in RoB2 under Domain 3. | Missing outcome data in parallel-CRTs can cause bias in inferences where the missingness is differential across treatment arms. | Missing outcome data in parallel-CRTs can cause bias in inferences where the missingness is differential across treatment arms. SW-CRTs might be at additional risk if they require a longer duration of follow-up, with hence greater chance of attrition. |
| Bias in measurement of the outcome | Studies in which the treatment status is known by those assessing outcomes might be at risk of bias because of (subconscious) assessments of outcomes being preferential in one treatment group. Outcomes which are objective (eg, mortality) will be at reduced risk of this bias. This bias is described in RoB2 under Domain 4. | CRTs with objective outcomes are unlikely to be at risk of bias due to unblinded assessment of outcomes. | SW-CRTs with objective outcomes are unlikely to be at risk of bias due to unblinded assessment of outcomes. |
| Bias in selection of the reported result | Studies which do not pre-specify the primary outcome, along with primary assessment time are at risk of selecting positive outcomes at the time of reporting. This bias is described in RoB2 under Domain 5. | CRTs which are pre-registered on a trials registration database with pre-specification of outcomes will be at low risk of bias due to selection of outcome for reporting. | SW-CRTs which are pre-registered on a trials registration database with pre-specification of outcomes will be at low risk of bias due to selection of outcome for reporting. SW-CRTs that are not registered or have may be multiple primary outcomes will be at risk of selective reporting of outcomes. |

Domains taken from the cluster extension to RoB2 [Eldridge 2016]; Interpretation of domains are consistent but not directly taken with the interpretations provided in RoB2.

**Appendix 1: Sample size calculation for the PITHIA trial**

Power calculations are based on 20 clusters. The intervention will be implemented across 5 steps; each step is four months in duration, with 4 clusters being randomly chosen to cross at each step. There are no planned transition periods within the study. The study has 5 sequences and 6 periods.

Estimates for the expected sample size and correlations were obtained routinely collected data [Ayorinde 2019]. We followed the methodology proposed by Hooper et al. (2016) and calculations were implemented using the RShiny app (https://clusterrcts.shinyapps.io/rshinyapp/). As the trial has two primary outcomes a Bonferroni correction was applied and hence a 2.5% significance level will be used. Both primary outcomes will test a superiority hypothesis. We consider the second primary outcome here only.

The second primary outcome is a continuous outcome. The estimated median cluster-period size is 8. As the trial will run for 6 periods, it is anticipated that the total sample size will be 960 (=6*20*8).The target effect size is a mean difference of 6 (SD=16.06).

The estimated within period intra-cluster correlation (ICC) and cluster autocorrelation (CAC) were calculated as 0.06 and 0.08 respectively. Comparisons of restricted log-likelihoods indicated a model with cluster and cluster by period effects was a better fit to the data compared with a model which allowed for exponential decay and so we assume a two period decay structure.

Under a SW-CRT design

Assuming an average cluster-period size of 8, this outcome will have 89% power to detect a mean difference of 6. This changes very little even if the CAC is as high 0.9. Under this design all 20 clusters will eventually be exposed to the intervention condition and half of the participants will receive the intervention condition (anticipated to be 960/2=480).

Under a parallel-CRT

The cluster size will be 8*6=48. Under an exchangeable correlation structure (ICC=0.06) the study has approximately 76% power. Under this correlation structure a power of 90% is not achievable however large the cluster is. Under this design only 10 clusters will be exposed to the intervention condition and half of the participants will receive the intervention condition (anticipated to be 960/2=480).

For a fixed cluster size of 48 (study duration 24 (=6*4) months), and under the same assumptions, 15 clusters per arm are needed to achieve 90% power. For a fixed cluster size of 8 (study duration 3 months), and under the same assumptions, 32 clusters per arm are needed to achieve 90% power.

Under a two-period decay correlation structure if the CAC is 0.08 with a cluster-period size of 24 (=48/2) the power is about 92%. If the CAC is 0.9 then the power is about 78%. The power available under a parallel-CRT is thus dependent on the assumed correlation structure.
